# Obstructive Sleep Apnea as a Hidden Cause of Dizziness: A Report of Two Non-obese Patients

**DOI:** 10.7759/cureus.84779

**Published:** 2025-05-25

**Authors:** Nanako Akiyama, Yusuke Suzuki, Tatsuki Tanaka, Hiroshi Ito, Ryo Ichibayashi

**Affiliations:** 1 Division of Emergency Medicine, Department of Internal Medicine, Toho University Sakura Medical Center, Chiba, JPN

**Keywords:** diagnostic algorithms, dizziness, non-obese patients, obstructive sleep apnea, polysomnography

## Abstract

Dizziness is a common complaint in emergency medicine. Algorithms such as the standing assessment (STANDING) and head impulse, nystagmus, and distortion tests (Head Impulse, Nystagmus, and Test of Skew (HINTS)) are used as "diagnostic aids" and "initial assessment tools". However, obstructive sleep apnea (OSA) is often excluded from these paradigms, despite evidence showing an association between OSA and dizziness.

We report two patients with unexplained dizziness in whom conventional evaluations were non-diagnostic. Both exhibited sleep-related symptoms, and simplified polysomnography revealed moderate OSA. Mandibular advancement devices led to symptom resolution.

OSA may underlie dizziness when standard assessments are inconclusive. Screening for sleep-related symptoms is essential. We suggest targeted OSA screening in patients with unexplained dizziness and relevant risk factors.

## Introduction

Dizziness encompasses a wide range of etiologies, including peripheral causes such as benign paroxysmal positional vertigo (BPPV), central causes such as cerebrovascular disorders and brain tumors, presyncope, imbalance, and psychogenic dizziness. Various algorithms for managing dizziness have been developed to assist in the structured assessment of these patients [1].

Recent studies have suggested a possible association between obstructive sleep apnea (OSA) and dizziness. Proposed mechanisms include vestibular dysfunction, autonomic dysregulation, and cerebellar changes due to recurrent hypoxia [2-5]. Individuals at high risk for OSA, particularly those with poor sleep quality or shortened sleep duration, have been reported to experience dizziness more frequently [6]. Szeto and Kesser investigated the relationship between dizziness, daytime somnolence, and sleep-disordered breathing in elderly individuals. They found that both daytime somnolence and sleep apnea were independently associated with dizziness, emphasizing the importance of including these conditions in the differential diagnosis of dizziness [7]. However, precise prevalence data on dizziness among patients with OSA, or vice versa, remain lacking. Notably, existing clinical algorithms for dizziness assessment do not include evaluation for OSA. This gap raises the possibility that some cases of dizziness may remain unexplained due to unrecognized sleep-disordered breathing.

At our emergency department, we routinely use the STANDING algorithm, Head Impulse, Nystagmus, and Test of Skew (HINTS), ABCD2 score, and Schellong test for evaluating patients with dizziness [8-11]. We present two cases of unexplained dizziness in which these evaluations were unremarkable. Further investigation revealed moderate OSA, and symptoms resolved with mandibular advancement device therapy. These cases highlight the importance of considering OSA in the differential diagnosis of dizziness when standard assessments are inconclusive.

## Case presentation

Case 1

A 66-year-old woman was transported to the emergency department with an eight-month history of pulsatile headaches upon awakening and floating-type vertigo exacerbated by positional changes upon rising. She had not sought medical attention during the previous eight months, as she did not consider the symptoms severe enough to warrant a visit. Her medical history was significant for gastric cancer, for which she had undergone total gastrectomy, and she was receiving intramuscular vitamin B12 injections every three months. She was not taking any regular medications and had no history of smoking or alcohol consumption. On physical examination, her height was 162 cm, and her weight was 54.5 kg, with a body mass index (BMI) of 20.8 kg/m².

On arrival, her vital signs were as follows: alert and oriented, body temperature 35.5°C, blood pressure 144/90 mmHg, heart rate 78 beats per minute, respiratory rate 18 breaths per minute, and oxygen saturation 98% on room air. Positional changes during physical examination induced floating-type vertigo and nausea; however, no nystagmus or other neurological abnormalities were observed. The HINTS examination was negative, and neither the Dix-Hallpike test nor the supine head roll test elicited nystagmus. Laboratory tests revealed dyslipidemia, but no anemia or electrolyte imbalances. Electrocardiography showed a normal sinus rhythm with a heart rate of 66 beats per minute and no abnormalities. The Schellong test was within normal limits. To exclude the central causes of vertigo, a brain magnetic resonance imaging (MRI) was performed. The scan showed no pathological findings in the cerebellum, brainstem, or vertebrobasilar arteries, ruling out central nervous system involvement. As both cardiac and central causes of vertigo were considered unlikely, a peripheral cause was suspected. However, otolaryngology consultation did not yield a definitive diagnosis. Given the pattern of symptoms, that is, onset upon awakening, associated headaches and fatigue, and daytime sleepiness, OSA was considered in the differential diagnosis. A simplified overnight polysomnography revealed an apnea-hypopnea index (AHI) of 19.5 events per hour, consistent with moderate OSA (Table 1).

**Table 1:** A simplified overnight PSG The recording demonstrated a total sleep time of 384 minutes, with an AHI of 19.5 events/hour, consistent with moderate obstructive sleep apnea. The ODI was 18.6 events/hour. While mean SpO₂ remained within normal limits (96%), the minimum SpO₂ dropped to 83%, and SpO₂ was below 90% for 1.3% of the recording time. Fifteen apneas and 110 hypopneas were observed, with the most prolonged apnea lasting 35 seconds. The average heart rate during sleep was 66 bpm, within the expected range. PSG: polysomnography; AHI: apnea-hypopnea index; ODI: oxygen desaturation index

Item	Results	Reference value
Total sleep time (min)	384	≥360 minutes (estimated in simplified PSG)
AHI (/hour)	19.5	<5 events/hour: normal; 5-14: mild; 15-29: moderate; ≥30: severe
ODI 3% (/hour)	18.6	<5 events/hour
Mean SpO₂ (%)	96	>95%
Minimum SpO₂ (%)	83	≥90%
Time SpO₂ <90% (%)	1.3	<2%
Number of apneas	15	Few or none (occasional events may occur in healthy individuals)
Number of hypopneas	110	Few or none
Longest apnea (sec)	35	<10 seconds
Mean heart rate (bpm)	66	50-80 bpm (during sleep)

Given the absence of obesity, the moderate severity of OSA, and favorable anatomical features, a mandibular advancement device was deemed an appropriate alternative to continuous positive airway pressure (CPAP) therapy and was subsequently initiated to enhance sleep quality. At a three-month follow-up, the patient reported significant improvement in daytime sleepiness and headaches, with complete resolution of vertigo symptoms. Based on this clinical course, the vertigo was attributed to OSA.

Case 2

A 59-year-old man was transported to the emergency department with complaints of dizziness upon awakening, episodes of transient vision loss, and dysarthria. His medical history was notable for hypertension, for which he was taking telmisartan. He had a height of 170 cm, a weight of 60 kg, and a BMI of 20.8 kg/m². He reported a history of daily alcohol consumption (360 mL of sake, approximately 43 grams of ethanol) and a smoking history of 10 cigarettes per day from ages 22 to 40; he is currently abstinent from smoking.

On arrival, his vital signs were as follows: alert and oriented, body temperature 36.9°C, blood pressure 173/112 mmHg, heart rate 95 beats per minute, respiratory rate 15 breaths per minute, and oxygen saturation 95% on room air. Physical examination revealed floating-type vertigo; however, the HINTS examination was negative, and both neurological abnormalities and dysarthria had resolved by the time of evaluation.

Laboratory tests showed no evidence of anemia or electrolyte imbalance, but untreated dyslipidemia was noted. Electrocardiography demonstrated normal sinus rhythm with a heart rate of 84 beats per minute. A brain MRI was performed to exclude central causes of vertigo. It revealed no pathological findings in the cerebellum, brainstem, or vertebrobasilar arteries, ruling out central nervous system involvement. The ABCD2 score was calculated as 4 (hypertensive, speech disorder without muscle weakness, duration 60 minutes or more). The patient was diagnosed with a transient ischemic attack (TIA), and antiplatelet therapy was initiated. After admission, the patient experienced no recurrence of dizziness or dysarthria and was discharged because a head MRI performed 24 hours later showed no new cerebral infarction. In the outpatient department, after discharge, further history-taking revealed episodes of nocturnal awakening and significant daytime sleepiness, raising suspicion for OSA. A full-night polysomnography (Table 2) showed an AHI of 16.7 events per hour, consistent with moderate OSA.

**Table 2:** A simplified overnight PSG The recording showed a total sleep time of 345 minutes, slightly below the estimated reference of 360 minutes. The AHI was 16.7 events/hour, indicating moderate obstructive sleep apnea. The ODI was 10.4 events/hour. Although the mean SpO₂ was high at 99%, the minimum SpO₂ dropped to 89%, slightly below the normal threshold. SpO₂ fell below 90% for only 0.1% of the time. Ninety-six apneas were recorded, with the most prolonged apnea lasting 61 seconds. Hypopneas were not recorded separately. The average heart rate during sleep was 66 bpm, within the normal range. PSG: polysomnography; AHI: apnea-hypopnea index; ODI: oxygen desaturation index

Item	Results	Reference value
Total sleep time (min)	345	≥360 minutes (estimated in simplified PSG)
AHI (/hour)	16.7	<5 events/hour: normal; 5-14: mild; 15-29: moderate; ≥30: severe
ODI 3% (/hour)	10.4	<5 events/hour
Mean SpO₂ (%)	99	>95%
Minimum SpO₂ (%)	89	≥90%
Time SpO₂ <90% (%)	0.1	<2%
Number of apneas	96	Few or none (occasional events may occur in healthy individuals)
Number of hypopneas	-	Few or none
Longest apnea (sec)	61	<10 seconds
Mean heart rate (bpm)	66	50-80 bpm (during sleep)

Treatment with a mandibular advancement device was initiated following the approach taken in the first case. At a one-month follow-up, the patient reported improvement in daytime sleepiness and nocturnal awakenings, with complete resolution of dizziness.

## Discussion

We present two cases of dizziness where the cause could not be identified using examination, application of the algorithms above, or MRI and no peripheral or central abnormalities were found. We also report on the process leading to the diagnosis of OSA and the effective treatment intervention.

In Case 1, no central or peripheral cause was found. In Case 2, the patient presented with transient neurological symptoms and was initially diagnosed with TIA. As the ABCD2 score was 4, antiplatelet drugs were administered, and the patient was hospitalized for treatment. Both cases were ultimately diagnosed with OSA, and appropriate treatment intervention was possible.

Several hypotheses have been proposed regarding the mechanism by which OSA causes dizziness. First, repeated hypoxia may cause structural damage to the brainstem and cerebellum, which are related to the vestibular system [2]. Second, intermittent hypoxia-induced nerve inflammation and imbalance in autonomic function may destabilize blood flow in brain regions that maintain balance, inducing dizziness [3,4]. Third, impaired cerebral blood flow autoregulation may lead to reduced cerebellum and inner ear perfusion, which may lead to reduced vestibular system function [5]. Furthermore, it has been suggested that white matter damage and hippocampal degeneration associated with OSA impair vestibular integration broadly, affecting spatial cognition and balance [2,5]. It has also been pointed out that chronic sleep disorders caused by OSA jeopardize the integration of balance [6].

In both Cases 1 and 2, OSA was suspected and diagnosed based on examining sleep-related symptoms. Case 1 presented with throbbing headache, floating dizziness, fatigue, and daytime sleepiness upon awakening, while Case 2 presented with dizziness upon awakening, nocturnal awakening, and daytime sleepiness. OSA is generally more prevalent in obese patients, but both instances had normal BMIs. In addition, Case 2 had hypertension, untreated dyslipidemia, transient visual disturbances on awakening, and dysarthria. Hypertension and dyslipidemia are known risk factors for OSA [12], and visual disturbances on awakening and dysarthria are considered symptoms of TIA. Still, OSA is also known to increase the risk of cerebrovascular events, suggesting an indirect association with these symptoms [13].

The critical point in this case is that the understanding of sleep-related symptoms through interviews led to the diagnosis. When the patient's main complaint is sleep-related symptoms, it is easy to keep OSA in mind, but when the patient visits the hospital for other symptoms, as in this case, OSA is easily overlooked. In particular, active screening is recommended for patients with TIA or hypertension, as in Case 2. As for the relationship between OSA and dizziness, as mentioned above, cerebral blood flow disorders and hypoxia may affect the vestibular system. Both cases were diagnosed with OSA by simple polysomnography, and dizziness and sleep-related symptoms improved with mandibular advancement device therapy. Therefore, OSA may at least be a factor that exacerbates symptoms and may have contributed to preventing the recurrence of TIA [14].

OSA is known to be more common in elderly patients, obese patients, men, and those accompanied by daytime somnolence and morning headaches [15,16]. Two cases were elderly and not obese, but experienced daytime sleepiness and early morning headaches. For this reason, active questioning about sleep-related symptoms is essential for diagnosis, and OSA should be considered in cases of dizziness with unclear etiology. However, current dizziness management algorithms do not include an evaluation for OSA [1]. Incorporating factors such as a positive ABCD2 score and the presence of sleep-related symptoms into the diagnostic flow may reduce the number of cases classified as dizziness of unknown origin and improve OSA detection rates. While a revision of the algorithm is warranted, clinicians must prioritize targeted OSA screening in patients with unexplained dizziness and relevant risk factors.

The limitations of this report are as follows. First, peripheral dizziness could not be completely ruled out, and the test was performed only once, so the evaluation of reproducibility and repeatability was insufficient. Second, the diagnosis of OSA was based on a one-night simple polysomnography test, which may be affected by fluctuations in nocturnal symptoms and test conditions. Further evaluation with a comprehensive polysomnography may be warranted to confirm the diagnosis. Third, the assessment of the effectiveness of treatment relied on subjective symptom improvement, and no quantitative evaluation was performed using objective indicators. Furthermore, since only two cases were reported, it is insufficient to show the causal relationship between OSA and dizziness, and future prospective studies and large-scale studies are necessary.

## Conclusions

In this report, OSA was identified in two patients with dizziness that could not be diagnosed using conventional algorithms, and symptoms improved with mandibular advancement device therapy. Although a potential link between OSA and dizziness has been suggested, awareness in emergency departments remains low, and screening opportunities are limited. Since current algorithms lack OSA assessment, clinicians may miss this diagnosis, especially when dizziness coexists with sleep-related symptoms or cardiovascular comorbidities. While large-scale data are limited, emerging evidence supports this association. We suggest targeted OSA screening in patients with unexplained dizziness and relevant risk factors, rather than immediate changes to existing algorithms. Incorporating OSA into emergency care frameworks may enhance diagnostic accuracy and patient outcomes.
